# Financial literacy among anaesthetists in an academic department in Johannesburg

**DOI:** 10.4102/jcmsa.v3i1.111

**Published:** 2025-03-31

**Authors:** Ayesha Noor Mohamed, Zainub Jooma

**Affiliations:** 1 Department of Anaesthesia and Pain Medicine, Royal Perth Hospital, Perth, Australia; 2 Department of Anaesthesia, Faculty of Health Sciences, University of the Witwatersrand, Johannesburg, South Africa; 3 Chris Hani Baragwanath Academic Hospital, Department of Anaesthesia, Johannesburg, South Africa

**Keywords:** anaesthesia, financial literacy, financial wellness, retirement planning, physician wellness

## Abstract

**Background:**

Financial literacy is an important skill required to navigate the complex financial landscape. Wealth, income, race, age, geographical location and level of education affect financial literacy levels in South Africa. The aim of this study was to assess financial literacy among anaesthetists at the Department of Anaesthesiology, University of the Witwatersrand (WITS) and to identify factors associated with better financial literacy.

**Methods:**

This prospective, cross-sectional study was conducted using an anonymised questionnaire adapted from the South African Social Attitudes Survey. A financial literacy score out of 100 was calculated using four domain scores (financial knowledge, financial planning, financial control and product choice and awareness).

**Results:**

A total of 184 anaesthetists were included. The mean financial literacy score was 72. Gender, age, relationship status and years of employment did not influence the mean financial literacy scores. The mean scores for the individual domains were 79 for financial control, 76 for financial planning, 51 for product choice and 82 for financial knowledge. Lower levels of financial preparedness were observed among younger and junior anaesthetists as well as anaesthetists who self-reported as mixed race or black ethnicity.

**Conclusion:**

Anaesthetists at the Department of Anaesthesiology, University of the Witwatersrand have high levels of overall financial literacy but do not display adequate preparedness in planning for a secure financial future. We suggest integration of continuous financial education into medical training, from undergraduate studies through to specialisation.

**Contribution:**

This study was the first to investigate financial literacy among anaesthetists in South Africa. It has highlighted the need for formal education on financial literacy to be incorporated into medical training.

## Introduction

Financial literacy is defined as ‘a combination of awareness, knowledge, skill, attitude and behaviour necessary to make sound financial decisions and ultimately achieve individual financial well-being’.^1^ Financial literacy has been shown to impact saving and investment behaviour, debt management and borrowing practices, wealth accumulation and planning for retirement.^2^ Higher financial literacy can reduce workplace anxiety and may contribute to improved health outcomes, fewer financial disputes and reduced family conflicts.^3,4^ Anaesthetists who are from backgrounds of higher financial literacy tend to have higher levels of productivity.^3,4^ Rising life expectancy places greater responsibility on individuals to manage their personal finances effectively, highlighting the need for adequate savings and comprehensive retirement planning. Financial markets are also becoming more complex and diverse, offering a wider range of financial products. This cumulative challenge requires greater skill when an individual is navigating their financial course.^2^

Despite South Africa boasting a sophisticated financial sector, income wealth inequality in South Africa is among the highest in the world.^5,6,7^ According to the National Income Dynamics Study (NIDS) and related research, financial literacy tends to be higher among individuals who are more educated and have higher incomes.^6^ Previous investigations have demonstrated substantial differences in financial literacy across racial groups, with white anaesthetists and Asian anaesthetists demonstrating higher financial literacy.^5,6^ Those residing in the Eastern Cape, Northern Cape, Mpumalanga and the North West provinces were found to have lower financial literacy. This disparity may reflect the socioeconomic and educational characteristics associated with the demographic makeup of these regions.^5^ Data from the South African Social Attitudes Survey (SASAS) suggest that a considerable portion of adults in South Africa are not sufficiently financially literate and find fiscal matters challenging.^7^

According to a publication by The Johns Hopkins Carey Business School, physicians complete their training with very little knowledge of business or finance.^8^ Furthermore, a study conducted by Ahmad et al. at the Washington University School of Medicine highlighted deficiencies in resident’s financial literacy.^9^ Increasing years of specialisation delays earning potential, placing doctors behind their contemporaries in other fields.^8^ The healthcare sector is becoming increasingly complex with more regulations and greater competition, making it more difficult to run a successful medical practice.^8^ In addition, the rising cost of medical education incurs a greater amount of debt at the end of training, influencing personal and professional decisions.^10,11^ Aside from discouraging doctors from general practice and towards higher paying specialities,^10,12^ educational debt also influences choices such as marriage, starting a family and purchasing a home. The perceived delay in the natural social trajectory of an individual can lead to frustration and burnout.^11^ Anaesthetists already experience high levels of burnout^13,14^ with a 21% rate of burnout reported among anaesthetists at the Department of Anaesthesiology, University of the Witwatersrand.^14^

Financial literacy among doctors remains low.^8,9^ A study carried out at the Washington School of Medicine found that residents had low financial literacy and investment-risk tolerance, high debt and deficits in financial preparedness.^9^ It has also been established in the available literature that among anaesthesiologists who retire later than expected, the second most common reason quoted was not being able to afford to retire.^15^ In addition, the coronavirus disease 2019 (COVID-19) pandemic has had a negative financial effect on medical practices. Physicians reported reducing working hours and incurring salary reductions early in the pandemic.^16^ To date, there are no studies that have examined financial literacy among anaesthetists in South Africa.

The aim of this study was to assess overall financial literacy among anaesthetists at the Department of Anaesthesiology, University of the Witwatersrand and to identify factors that predict higher or lower levels of financial literacy.

## Research methods and design

This prospective, contextual and descriptive study was conducted between the months of October 2020 and January 2021 at the Department of Anaesthesiology, University of the Witwatersrand. The study sample consisted of 238 anaesthetists working in the department and included interns, medical officers, registrars and consultants.

The research tool consisted of an anonymised, self-administered questionnaire adapted from the original version developed by the Human Sciences Research Council (HSRC) in collaboration with the Financial Services Board.^7^ Permission was obtained prior to its use and adaptation. The questionnaire was based on work conducted by the Organisation for Economic Co-operation on financial literacy. This questionnaire has been revised by the HSRC in five studies since the period of its development to track the progression of financial literacy in South Africa.^7,17,18,19^ The questionnaire is available in Online Appendix 1. It is a multi-dimensional tool and tests four major domains: financial control, financial planning, product choice and awareness and financial knowledge.

The questionnaire consisted of 38 questions including ascertaining five demographic criteria, that is age, gender, race, years of employment and marital status. The adaptations to the original questionnaire were made only to the demographic questions where province, living standard and geographical location were excluded as variables, and years of employment was included. Respondents were given fixed categories to choose from in the five demographic questions, rather than providing an empty box for a written answer. Race was categorised in alignment with the SASAS study as follows: Black anaesthetists, white anaesthetists, Indian anaesthetists, mixed race anaesthetists or other. The questionnaire was distributed to anaesthetists working in the department through electronic mail using a REDcap^20^ survey link.

Data were analysed and reported in congruence with the SASAS study. Data were analysed using the IBM^®^ Statistical Package for Social Sciences (SPSS) statistics software, version 27.0. Descriptive statistics were reported including frequencies and percentages and means and standard deviations (s.d.). To identify associations between mean scores in the financial literacy domains and demographics, the Mann–Whitney, Kruskal–Wallis *t*-test and analysis of variance (ANOVA) tests were conducted depending on the normality of the data. For comparison of demographic characteristics across individual questions, the Pearson chi-square and Fisher’s exact tests were used. Results were reported using odds ratios (OR) and 95% confidence intervals (CIs). A *p*-value of < 0.05 was considered statistically significant. Incomplete questionnaires were included in the data analysis, resulting in a small amount of missing data.

### Ethical considerations

The questionnaire was distributed, using a REDcap^20^ public survey link, to anaesthetists working in the department during the study period. Ethics approval was obtained from the University of the Witwatersrand Human Research Ethics Committee (M200626) and other relevant authorities. Consent was implied upon completion of the questionnaire. The anaesthetists’ email addresses were de-linked from the questionnaire to ensure anonymity. The questionnaires were scored using the same weighting system that was used in the SASAS study.

## Results

Of the 238 surveys sent out to eligible participants, 54 were not returned. The remaining 184 responses from anaesthetists were included in the study. Demographic characteristics are summarised in Table 1.

**TABLE 1:** Demographic characteristics of anaesthetists.

Demographic	*n*	%
**Gender**		
Male	70	38.0
Female	114	62.0
**Marital status**		
Married	113	61.4
Unmarried	71	38.6
**Age (years)**		
16–24	1	0.5
25–34	108	58.7
35–49	59	32.1
50–64	10	5.4
> 65	5	2.7
Missing	1	0.5
**Race**		
Black anaesthetists	47	25.5
White anaesthetists	89	48.4
Indian anaesthetists	34	18.5
Mixed race anaesthetists	5	2.7
Other	8	4.3
Missing	1	0.5
**Years employed**		
0–3	21	11.4
4–6	37	20.1
7–9	47	25.5
10–12	31	16.8
> 12	47	25.5
Missing	1	0.5

**FIGURE 1:**
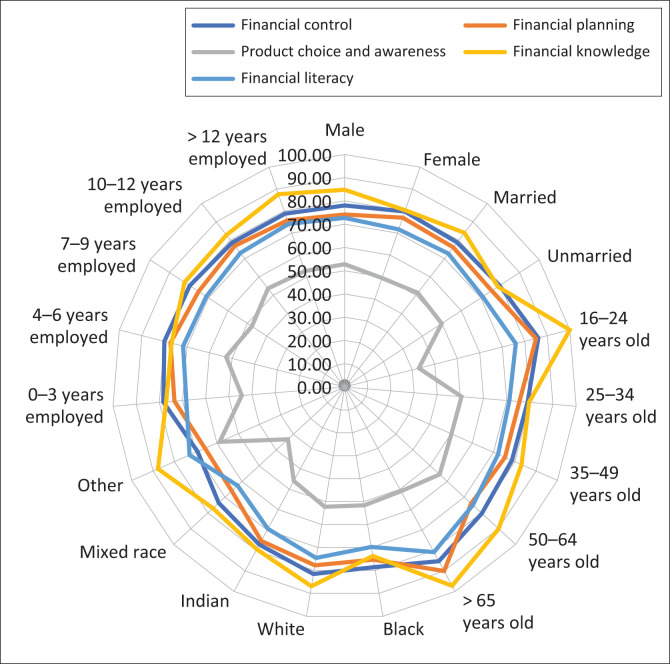
Demographic distribution of mean financial literacy and domain scores.

The mean financial literacy score on a 0–100 scale was 72. The results for individual domains indicated a mean score of 79 for financial control, 76 for financial planning, 51 for product choice and 82 for financial knowledge. The demographic distribution of mean scores is represented graphically in Figure 1.

With regard to mean financial literacy, those who self-reported as white anaesthetists scored significantly higher (mean = 74.55, s.d. = 9.179) than those who identified as mixed race (mean = 62.60, s.d. = 15.372), *p* = 0.049, Cohen’s *d* = 1.253. No significant differences were observed across other demographic categories.

Statistically significant differences in domain mean scores across demographic categories were only found in the financial knowledge domain with those who were married scoring higher than those who were unmarried (*p* = 0.034); those over the age of 65 years scoring higher than those aged between 16–34 years and 35–49 years (*p* = 0.005 and *p* = 0.012, respectively); those who self-reported as white anaesthetists scoring higher than those who self-reported as black anaesthetists or Indian anaesthetists (*p* < 0.0005 and *p* = 0.018, respectively) and those who were employed > 12 years scoring higher than those who were employed for 0–3 years and 4–6 years (*p* = 0.007 and *p* = 0.009, respectively).

Financial behaviours and attitudes were examined. The majority of anaesthetists (62%) reported having a household budget. An analysis of demographic characteristics demonstrated that Indian anaesthetists and ‘other’ anaesthetists were significantly less likely to have a household budget (*p* = 0.026), with OR of 0.314 (95% CI = 0.12–0.82) for Indian anaesthetists and 0.105 (95% CI = 0.02–0.60) for ‘other’ anaesthetists.

When asked to rate their agreement (on a five-point scale) to the statement ‘money is there to be spent’, 79.9% anaesthetists disagreed with the statement compared to the 20.1% who agreed (*p* < 0.0005). It was observed that 65% of anaesthetists set long-term financial goals and are committed to achieving them. When asked if, before buying something, they carefully considered if they could afford it, 74% responded that they do. When asked if they pay their bills on time, 83% responded that they always do.

Financial decision making was examined. When asked who was responsible for day-to-day money management decisions in the household, 98% responded that they were responsible either by themselves or together with someone else. Anaesthetists were asked to rate their confidence in buying financial services and products without the help of a financial adviser and only 35% indicated confidence. When asked if they always research their financial decisions thoroughly, 69% of anaesthetists agreed that they do. The most influential factor impacting financial decision making was ‘advice from a financial adviser’. Other sources of learned financial knowledge included parents and spouses. The share of those who felt that they possessed an unsuitable financial product in their portfolio was 40%, while 36% of anaesthetists experienced regret regarding one or more financial decisions. The demographic characteristics associated with financial regrets are outlined in Table 2 where race, gender, marital status and years employed were significantly associated.

**TABLE 2:** Demographic characteristics associated with financial regret and shortfall.

Indicator: Financial regret in managing	Demographic	Yes		No		*p*-value	OR	95% CI
		*n*	%	*n*	%			
Savings or investments	**Gender:**	-	-	-	-	0.007**	-	-
	Male	17	60.7	53	34.0	-	3.003	1.31–6.87
	Female	11	39.3	103	66.0	-	1.000	-
	**Marital status:**	-	-	-	-	0.009**	-	-
	Married	11	39.3	102	65.4	-	1.000	-
	Unmarried	17	60.7	54	34.6	-	2.919	1.28–6.68
Tax	**Gender:**	-	-	-	-	0.004**	-	-
	Male	15	65.2	55	34.2	-	3.614	1.44–9.05
	Female	8	34.8	106	65.8	-	1.000	-
Managing credit and debt	**Race:**	-	-	-	-	0.049*	-	-
	Black anaesthetists	12	37.5	35	23.2	-	1.000	-
	White anaesthetists	12	37.5	77	51.0	-	0.455	0.19–1.11
	Indian anaesthetists	4	12.5	30	19.9	-	0.389	0.11–1.33
	Mixed race anaesthetists	3	9.4	2	1.3	-	4.375	0.65–29.41
	Other	1	3.1	7	4.6	-	0.417	0.05–3.74
Experienced a financial shortfall	**Race:**	-	-	-	-	< 0.0005	-	-
	Black anaesthetists	26	44.1	21	17.1	-	1.000	-
	White anaesthetists	23	38.9	65	52.8	-	0.286	0.14–0.60
	Indian anaesthetists	5	8.5	29	23.6	-	0.138	0.05–0.42
	Mixed race anaesthetists	3	5.1	2	1.6	-	1.212	0.19–7.94
	Other	2	3.4	6	4.9	-	0.269	0.05–1.47
	**Years employed:**	-	-	-	-	0.024**	-	-
	0–3	4	6.8	17	13.8	-	0.486	0.14–1.70
	4–6	18	30.5	19	15.4	-	1.958	0.80–4.78
	7–9	9	15.3	38	30.9	-	0.489	0.19–1.27
	10–12	13	22.0	18	14.6	-	1.493	0.58–3.83
	> 12	15	25.4	31	25.2	-	1.000	-

OR, odds ratios; CI, confidence interval.

** , Pearson’s chi-square;

* , Fisher’s exact test.

Anaesthetists were questioned as to whether they experienced a financial shortfall in the last 12 months, to which 33% responded yes. The significant demographic characteristics associated with a financial shortfall are outlined in Table 2 with more black and mixed race anaesthetists and more junior anaesthetists, having experienced a financial shortfall. When asked about strategies to cope with a financial shortfall, 78% of anaesthetists either draw money out of a savings account or transfer money from a savings to a current account instead of using credit facilities.

Saving behaviour was reflected in anaesthetists’ ability to plan for financial emergencies, saving practices and retirement planning. An emergency fund that could cover 3 months of expenses was held by 75% of anaesthetists. When questioned about the ways in which they had been actively saving financial resources in the last 12 months, 76% transferred money into a savings account, 48% allowed money to accumulate in a bank account and 55% were purchasing financial investments and products (excluding pension funds). Only 8.2% of anaesthetists were entirely confident in their retirement planning. A retirement annuity was held by 82%, a provident fund held by 24% and a pension fund held by 54% of anaesthetists. The demographic characteristics affecting saving practices and retirement planning are summarised in Table 3, where race, gender, age and years of employment all have had a significant association.

**TABLE 3:** Demographic characteristics affecting saving practices and retirement planning.

Indicator: Savings	Demographic	Yes		No		*p*-value	OR	95% CI
		*n*	%	*n*	%			
Purchased financial investment products as a form of saving	**Race:**	-	-	-	-	0.001*	-	-
	Black anaesthetists	15	14.9	32	39.0	-	1.000	-
	White anaesthetists	60	59.4	29	35.4	-	4.414	2.07–9.41
	Indian anaesthetists	20	19.8	14	17.1	-	3.048	1.22–7.63
	Mixed race anaesthetists	1	0.9	4	4.9	-	0.533	0.06–5.19
	Other	5	5.0	3	3.7	-	3.556	0.75–16.87
Holds a retirement annuity	**Gender:**	-	-	-	-	0.031**	-	-
	Male	52	34.4	18	54.5	-	1.000	-
	Female	99	65.6	15	45.5	-	2.285	1.07–4.90
	**Years employed:**	-	-	-	-	0.001**	-	-
	0–3	11	7.3	10	30.3	-	1.000	-
	4–6	34	22.7	3	9.1	-	10.303	2.40–44.29
	7–9	36	24.0	11	33.3	-	2.975	1.00–8.85
	10–12	28	18.7	3	9.1	-	8.485	1.96–36.78
	> 12	41	27.3	6	18.2	-	6.212	1.85–20.86
Holds a provident fund	**Age (years):**	-	-	-	-	0.041*	-	-
	16–34	23	52.3	86	61.9	-	1.000	-
	35–49	13	29.5	46	33.1	-	1.057	0.49–2.28
	50–64	6	13.6	4	2.9	-	5.609	1.46–21.55
	> 65	2	4.5	3	2.2	-	2.493	0.39–15.81
Holds a pension fund	**Age† (years):**	-	-	-	-	< 0.0005**	-	-
	16–34	42	42.9	67	78.8	-	1.000	-
	35+	56	57.1	18	21.2	-	4.963	2.57–9.57
	**Years employed:**	-	-	-	-	< 0.0005**	-	-
	0–3	3	3.1	18	21.2	-	1.000	-
	4–6	17	17.3	20	23.5	-	5.100	1.28–20.32
	7–9	18	18.4	29	34.1	-	3.724	0.96–14.46
	10–12	20	20.4	11	12.9	-	10.909	2.62–45.43
	> 12	40	40.8	7	8.2	-	34.286	7.94–147.99

OR, odds ratios; CI, confidence interval.

** , Pearson’s chi-square;

* , Fisher’s exact test.

† , Categories with similar results were condensed for ease of reporting.

With regard to investment products, 50% of anaesthetists owned unit trusts, 42% owned shares in the stock exchange, 10% held educational policies and 65% held investment or saving policies. Demographic characteristics affecting investment behaviour are outlined in Table 4, where race and gender were found to significantly affect investment behaviour. Short-term insurance products, such as home-owners insurance or household contents insurance were held less by those who were young and unmarried. Long-term insurance products (life insurance, medical aid or disability cover) were also held significantly less by those employed 0–3 years. Table 4 outlines the demographic characteristics affecting and owning long-term insurance products.

**TABLE 4:** Demographic characteristics affecting investment behaviour and long-term insurance products.

Indicator: Investments	Demographic	Yes		No		*p*-value	OR	95% CI
		*n*	%	*n*	%			
Holds unit trusts	**Race:**	-	-	-	-	0.027*	-	-
	Black anaesthetists	17	18.9	30	32.3	-	1.000	-
	White anaesthetists	48	53.3	41	44.1	-	2.066	0.10–4.27
	Indian anaesthetists	20	22.2	14	15.1	-	2.521	1.02–6.24
	Mixed race anaesthetists	0	0.0	5	5.4	-	-	-
	Other	5	5.6	3	3.2	-	2.941	0.62–13.90
Holds Educational policies	**Gender:**	-	-	-	-	0.002**	-	-
	Male	1	5.3	69	41.8	-	1.000	-
	Female	18	94.7	96	58.2	-	12.937	1.69–99.23
	**Race:**	-	-	-	-	0.025*	-	-
	Black anaesthetists	10	55.6	37	22.4	-	1.000	-
	White anaesthetists	4	22.2	85	51.5	-	0.174	0.05–0.59
	Indian anaesthetists	3	16.7	31	18.8	-	0.358	0.09–1.42
	Mixed race anaesthetists	1	5.6	4	2.4	-	0.925	0.09–9.22
	Other	0	0.0	8	4.8	-	-	-
Holds shares on the stock exchange	**Race:**	-	-	-	-	0.015*	-	-
	Black anaesthetists	11	14.1	36	34.3	-	1.000	-
	White anaesthetists	46	59.0	43	41.0	-	3.501	1.58–7.74
	Indian anaesthetists	16	20.5	18	17.1	-	2.909	1.12–7.55
	Mixed race anaesthetists	1	1.3	4	3.8	-	0.818	0.08–8.10
	Other	4	5.1	4	3.8	-	3.273	0.70–15.29
Holds life insurance	**Years employed:**	-	-	-	-	0.001**	-	-
	0–3	9	6.4	12	28.6	-	1.000	-
	4–6	31	22.0	6	14.3	-	6.889	2.02–23.55
	7–9	35	24.8	12	28.6	-	3.889	1.31–11.51
	10–12	27	19.1	4	9.5	-	9.000	2.31–35.07
	> 12	39	27.7	8	19.0	-	6.500	2.06–20.56
Holds medical Aid	**Years employed:**	-	-	-	-	0.035*	-	-
	0–3	14	8.8	7	29.2	-	1.000	-
	4–6	32	20.1	5	20.8	-	3.200	0.87–11.84
	7–9	40	25.2	7	29.1	-	2.857	0.85–9.60
	10–12	30	18.9	1	4.2	-	15.000	1.68–133.92
	> 12	43	27.0	4	16.7	-	5.375	1.37–21.12
Holds disability cover	**Race:**	-	-	-	-	0.007*	-	-
	Black anaesthetists	33	23.7	14	31.8	-	1.000	-
	White anaesthetists	75	54.0	14	31.8	-	2.273	0.98–5.30
	Indian anaesthetists	23	16.5	11	25.0	-	0.887	0.34–2.30
	Mixed race anaesthetists	1	0.7	4	9.1	-	0.106	0.01–1.04
	Other	7	5.0	1	2.3	-	2.970	0.33–26.44
	**Years employed:**	-	-	-	-	0.002**	-	-
	0–3	9	6.5	12	27.2	-	1.000	-
	4–6	27	19.4	10	22.7	-	3.600	1.17–11.13
	7–9	36	25.9	11	25.0	-	4.364	1.46–13.07
	10–12	26	18.7	5	11.4	-	6.933	1.91–25.18
	> 12	41	29.5	6	13.6	-	9.111	2.70–30.77

OR, odds ratios; CI, confidence interval.

** , Pearson’s chi-square;

* , Fisher’s exact test.

Basic arithmetic, knowledge on interest and inflation and basic financial acumen were tested. Basic arithmetic questions were answered correctly by the majority of anaesthetists. A question on inflation was answered incorrectly by 57% of anaesthetists. A question on interest was answered incorrectly by 28% of anaesthetists. The demographic characteristics affecting knowledge on interest and inflation are outlined in Table 5. Self-rated knowledge on financial matters was indicated to be ‘about average’ by 50%, ‘quite low’ by 24% and ‘very low’ by 11% of anaesthetists.

**TABLE 5:** Demographic characteristics affecting knowledge on interest and inflation.

Indicator: Knowledge	Demographic	Correct		Incorrect		*p*-value	OR	95% CI
		*n*	%	*n*	%			
Inflation question	**Race:**	-	-	-	-	0.001*	-	-
	Black anaesthetists	11	14.1	36	34.3	-	1.000	-
	White anaesthetists	51	65.4	38	36.2	-	4.392	1.98–9.73
	Indian anaesthetists	10	12.8	24	22.9	-	1.364	0.50–3.71
	Mixed race anaesthetists	2	2.6	3	2.9	-	2.182	0.32–14.77
	Other	4	5.1	4	3.8	-	3.273	0.70–15.29
	**Gender:**	-	-	-	-	0.002**	-	-
	Male	40	50.6	30	28.6	-	1.000	-
	Female	39	49.4	75	71.4	-	0.390	0.21–0.72
	**Age† (years):**	-	-	-	-	0.006**	-	-
	16–34	40	51.3	69	65.7	-	1.000	-
	35–49	26	33.3	33	31.4	-	1.359	0.71–2.59
	50+	12	15.4	3	2.9	-	6.900	1.84–25.93
Interest question	**Race:**	-	-	-	-	0.021*	-	-
	Black anaesthetists	27	20.3	20	40.0	-	1.00	-
	White anaesthetists	71	53.4	18	36.0	-	2.922	1.35–6.35
	Indian anaesthetists	26	19.5	8	16.0	-	2.407	0.90–6.42
	Mixed race anaesthetists	2	1.5	3	6.0	-	0.494	0.08–3.24
	Other	7	5.3	1	2.0	-	5.185	0.59–45.58

OR, odds ratios; CI, confidence interval.

** , Pearson’s chi-square;

* , Fisher’s exact test.

† , Categories with similar results were condensed for ease of reporting.

## Discussion

Mean financial literacy among anaesthetists at the Department of Anaesthesiology, University of the Witwatersrand is far higher than the average South African’s score, which is 54 (0–100 scale).^7,17,18,19^ The variation across demographic groups for mean financial literacy scores is not wide. Gender, age, relationship status and years of employment did not inform any enduring trend with regard to mean financial literacy scores. This is inconsistent with the finding in the SASAS study that found white and Indian South Africans scored relatively higher than their black and mixed race counterparts, those who were older scored higher than their younger counterparts and those who were married scored higher than those who were unmarried.^7^ According to the SASAS study and the NIDS study, gender did not play a role in affecting financial literacy.^6,17,18,19^ These findings strengthen the observation that educational attainment is one of the key factors driving overall financial literacy in South Africa.^7^

Further analysis of anaesthetists’ financial behaviour indicated that they generally are fiscally responsible, engage in saving practices, are actively involved in household financial management and conduct research prior to making financial decisions. This indicates good financial control, which is consistent with the average trend of financial control among employed and educated adult South Africans.^7^ This aligns with prior investigations conducted in high-income countries, which demonstrated that medical doctors tend to be aware of and involved in their personal financial matters.^9,21^ Financial knowledge among anaesthetists is above average in South Africa, with most of them being able to understand basic arithmetic, interest and general financial concepts. This is in keeping with the SASAS studies where educational attainment was found to be associated with a higher financial knowledge score.^7^ Over half of the anaesthetists did not understand the concept of inflation, as it was presented in the questionnaire, indicating a crucial gap in knowledge. Within the financial knowledge domain, it was also found that those who self-reported as married, older, white anaesthetists and employed for longer scored higher than their counterparts. This is inconsistent with the SASAS findings where it was found that younger anaesthetists have generally higher knowledge scores.^7^

Despite having good financial control and knowledge, anaesthetists display a lack of confidence in their ability to manage their financial portfolios and plan for their retirement by themselves (without the help of a financial adviser). Studies involving medical doctors in the United States yielded similar results, although they also demonstrated a tendency to distrust and underutilise financial advisers.^9,21^ In contrast, this study indicates that anaesthetists frequently rely on financial advisers to inform their financial decisions. A large proportion of anaesthetists also expressed experiencing financial regret. This could be explained by the similarity in income among respondents as the SASAS study described higher financial regret among higher income groups.^17^ A large proportion of anaesthetists in this study indicated not investing in diverse financial products such as unit trusts, shares or investment policies, instead, allowing funds to accumulate in bank and savings accounts. This is possibly because of risk aversion, as observed in previous similar investigations.^9^

Younger age and more junior anaesthetists indicated fewer investments, did not hold long-term insurance products such as disability cover, medical aid and life insurance and were not utilising retirement annuities, pension funds or provident funds. This finding is consistent with the SASAS report that life-cycle efforts have a clear impact on financial planning.^7^ This practice is ill-advised because doctors begin their formal medical career at a disadvantage due to the length of their degree, educational debt and increased years of specialisation.^8,10^ It is, therefore, essential that they avoid a delay in financial planning at the beginning of their formal employment and begin employment with an established plan in place. It was also found that black and mixed race anaesthetists (as compared to their white and Indian counterparts) reported more financial shortfall; more regret regarding credit and loans; invested less in unit trusts and shares on the stock exchange and invested less in financial products as a form of active saving. This has been an enduring trend throughout the literature regarding financial literacy as South Africa has had a continuous struggle with economic inequality since the end of the apartheid period.^5,6,7^ Unemployment and lack of access to higher education, while improving, still have an effect on the economic growth and empowerment of some population groups.^7^ This study, being quantitative in nature, did not investigate the individual social backgrounds of each anaesthetist participating in the study. It is important to keep this in mind when interpreting the results as these circumstances would also affect certain results. The small number of self-reported mixed race anaesthetists makes it difficult to draw firm conclusions in this group about the findings in this study. This is also evident from the wide CIs found in many of the comparisons.

Financial resources are among the most essential assets an individual accumulates and are regarded as a significant source of stress for employees.^3^ Anaesthetists are known to experience high levels of burnout;^14^ therefore, equipping them with tools to plan a secure financial future will alleviate the stress of financial concerns. The current literature suggests that a comprehensive financial literacy framework requires a course on personal finance to be delivered at three separate times during a doctor’s training, that is during medical school, residency and fellowship training. In addition to that, an 8-h to 10-h course on business finance should also be delivered.^8^ A dedicated curriculum should be developed to teach anaesthetists practically and from a young age about active saving, investing, retirement planning and prudent financial practices. Anaesthetists also need to be continuously educated about financial concepts such as inflation, compounding interest, risk diversification and tax. Financial education should encourage timely planning prior to graduation, with an emphasis on implementing financial plans upon entering professional practice.

This study has several strengths. The study was conducted in congruence with the SASAS studies, which are reliable and reproducible studies performed on a nationwide scale in South Africa.^7,17,18,19^ For this reason, some of the data garnered from this study may be reliably compared to the data that already exists in South Africa regarding financial literacy. Inferential and bivariate analyses conducted on specific sections enhance the study’s rigour, enabling more precise conclusions about demographic distributions across various areas.

The use of convenience sampling in this study presents a limitation, as all anaesthetists shared similar educational and employment backgrounds and were from a single department within one specialised field. Consequently, these results may not be generalisable to broader populations. This study differs from the SASAS study, which utilised larger, more diverse samples and applied a wider, more advanced range of statistical analyses.^7,17,18,19^ The reliance on self-reported knowledge introduces potential over or under-estimation of financial knowledge by participants in this study. The participants could also be prone to social desirability bias, where respondents adjust their answers to suit socially acceptable ones. Finally, the study did not deeply analyse the individual social backgrounds of the respondents. This could be a potential area for further study.

## Conclusion

Anaesthetists at the Department of Anaesthesiology, University of the Witwatersrand demonstrated above-average financial literacy compared to the general South African population. However, participants reported low levels of financial confidence and preparedness. No specific demographic category was associated with a lower mean financial literacy score; however, younger and less experienced anaesthetists showed less effective financial planning behaviour and suboptimal financial product choices. We recommend integrating ongoing financial education into medical training, from undergraduate studies through to specialisation.
